# The Effect of Conductive Polyaniline on the Anti-Fouling and Electromagnetic Properties of Polydimethylsiloxane Coatings

**DOI:** 10.3390/polym15132944

**Published:** 2023-07-04

**Authors:** Yarui Guo, Yuhong Qi, Chen Zhang, Shukun Zhang, Zhanping Zhang

**Affiliations:** Department of Materials Science and Engineering, Dalian Maritime University, Dalian 116026, China; gyr1998@dlmu.edu.cn (Y.G.); pudding@dlmu.edu.cn (C.Z.); zsk@dlmu.edu.cn (S.Z.); zzp@dlmu.edu.cn (Z.Z.)

**Keywords:** antifouling, electromagnetic performance, coating, PANI, PDMS

## Abstract

In this paper, four conductive polyaniline powders doped in hydrochloric acid, sulfuric acid, phosphoric acid, and sulfonic acid were selected and blended with polydimethylsiloxane to prepare coatings with an electromagnetic absorption effect and fouling desorption effect, respectively. A UV spectrophotometer was used to evaluate the settling rate of the powders. Fourier transform infrared spectrometry, laser confocal microscopy, and scanning electron microscopy were used to observe the morphology and structure of the powder and the coating. The interface properties of the coatings were characterized using a contact angle measurement, the mechanical properties of the coatings using a tensile test, and the electromagnetic properties of the powders and microwave absorption properties of the coatings using vector network analyzers. Meanwhile, the antifouling performance of the coatings was evaluated via the marine bacteria adhesion test and benthic diatom adhesion test, and the effect of conductive polyaniline on the antifouling performance of the coating was analyzed. The results show that adding polyaniline reduced the surface energy of the coating and increased the roughness, mechanical properties and anti-fouling properties of the coating. Moreover, adding appropriate polyaniline powder can enhance the electromagnetic wave loss of the coating. The followings values were recorded for a hydrochloric-acid-doped polyaniline coating: lowest surface energy of 17.17 mJ/m^2^, maximum fracture strength of 0.95 MPa, maximum elongation of 155%, maximum bandwidth of 3.81 GHz, and peak of reflection loss of −23.15 dB. The bacterial detachment rate of the polydimethylsiloxane (PDMS) samples was only 30.37%. The bacterial adhesion rates of the composite coating containing hydrochloric-acid-doped polyaniline were 4.95% and 2.72% after rinsing and washing, respectively, and the desorption rate was 45.35%. The chlorophyll concentration values were 0.0057 mg/L and 0.0028 mg/L, respectively, and the desorption rate was 54.62%.

## 1. Introduction

In 1985, the use of polyaniline (PANI) became widespread for corrosion protection. DeBerry [1] first suggested that conductive PANI coatings have anticorrosive properties. A conductive PANI coating was deposited on stainless steel, and electron transfer occurred between the coating and the passive metal, demonstrating that PANI plays a similar role to anode protection. The corrosion protection that the conductive polymer provides to metal comes in the following three forms. (1) PANI can be used directly as anti-corrosion coating, but the density of the conductive polymer film is poor, and the shielding effect of the corrosive medium is not ideal [2,3]. (2) PANI can be used as a corrosion inhibitor to protect metals. (3) PANI can be mixed with traditional polymers; this method can solve the problem of the limited conductive polymer shielding effect on corrosive media and also has a reduced cost [4,5]. Zhang et al. [6] conducted a comparative study on the anticorrosive properties of PANI coatings containing different ring substituents, and found that the effect of the substituents on the anticorrosive properties of the coatings was mainly reflected in their hydrophilicity/hydrophobicity, and the hydrophobic substituents increased the contact angle of the coatings, which made the coatings form an effective barrier to the penetration of corrosive media, enhancing the anticorrosive properties of the coatings. N atoms in the PANI molecular chain have unshared electron pairs [7]; provided that the metal atoms have empty orbitals, the two can form coordination bonds and adsorb firmly on the surface of metal substrates, forming a protective film and reducing the corrosion rate of metal substrates. Sathyanara [8] studied the development of an anti-corrosion coating with phosphoric-acid-doped PANI and an epoxy base material. The corrosion resistance of polymer-coated steel can protect steel in a certain concentration of salt water or acidic substances. Evidence has shown that PANI has excellent anti-corrosion properties in a variety of environments.

Most of the research on PANI is focused on the study of corrosion protection mechanisms, such as the electrochemical corrosion protection of steel and other metals. However, there is some controversy about the actual corrosion protection effect of coatings prepared from PANI materials. Secondly, application research into PANI has not included it in the concept of heavy anti-corrosion systems, and research into anti-corrosion systems featuring polyaniline coatings, which provide coatings with a monolithic function, is currently lacking. In this context, Shahhosseini et al. [9,10] successfully synthesized poly (4-(2-Furyl) benzenamine) (PFBA) and poly (4-(2-Thienyl) benzenamine) (PTBA) coatings doped with dodecyl benzene sulphonate. Compared with PANI, these coatings exhibit better corrosion protection on steel electrodes and have outstanding potential in corrosion resistance. Bandeira et al. [11] studied the corrosion resistance of PANI and polyvinyl chloride (PVC) co-blended polymers on carbon steel, and the results show that a non-conductive PVC coating offers protection by acting as a physical barrier to electrolyte permeation. The blended coatings offer corrosion protection through a combination of these passivation and barrier effects. In 1999, Wang et al. [12] carried out a preliminary study on the antifouling performance of a polyaniline/epoxy resin anticorrosive coating. The study, which was conducted in the southern sea area of China, found that a coating composed of conductive polyaniline and epoxy resin can last for 6 months, that is, during this period, the fouling rate on the coating is less than 10%. In addition, the results of the panel test in sea water show that the coating containing doped polyaniline (ES) exhibits a certain antifouling effect, while the coating containing intrinsic-state polyaniline did not show a significant antifouling effect [12]. The antibacterial and antifouling mechanism of the ES [13,14] consists of two main aspects: (1) the electric charge effect. The ES structure contains N^+^, which can interact with the negative charge carried by the bacterial surface, leading to the rupturing of the cell wall/cell membrane in the bacterial structure, causing bacterial inactivation and (2) conductivity. The ES has excellent electrical conductivity. A weak current is applied to the surface of the polyaniline composite coating so that the polyaniline electrolyzes the Cl^−^ in seawater into ClO^−^, thereby preventing biological contamination.

Baldissera et al. [13] experimentally investigated how doped polyaniline (PANI-ES and PANI-DBSA) can be used as an additive in antifouling coatings in the sulfonated state (SPAN), which can greatly reduce the amount of cuprous oxide in coatings, and thus reduce environmental pollution. This may be due to the reaction between cuprous ion and polyaniline, which produces copper ions, an active biocide that is beneficial for the prevention of marine pollution. Hou et al. [14] studied the one-step synthesis of silver/polyaniline nanocomposites and applied them to antimicrobial agents. The antibacterial performance of the composites was evaluated via live cell counting of Gram-negative *Escherichia coli, Staphylococcus aureus* and fungal yeast. The composites were found to have enhanced antibacterial efficacy and the following proposed antibacterial mechanism. The interaction of silver nanoparticles with polyaniline changed the structure and morphology of both composites, and polyaniline effectively stabilized the silver nanoparticles to avoid their aggregation in the composites.

At present, polyaniline/epoxy resin and polyaniline/acrylic resin anti-corrosion and anti-fouling coatings are in a phase of rapid development into environmentally friendly anti-fouling coatings [15,16], while polyaniline/silicone-resin-related research is lacking, as is research related to most hull anti-fouling and absorbing coatings. Future trends in development will transform these coatings from only having anti-corrosion and antifouling functions to undergoing multi-functional integration.

Polyaniline, as a typical conductive polymer, is the subject of extensive research on electromagnetic shielding [17,18]. Unlike traditional electromagnetic shielding materials, it is light and cheap and does not degrade the mechanical properties of the material. Faez et al. [19] prepared and investigated a conductive ternary compound based on nitrile rubber, Ethylene-propylene-diene monomer rubber (EPDM) and polyaniline with excellent microwave absorption properties in the frequency range from 8 GHz to 12 GHz. Zhang et al. [20] studied the microwave radiation absorption properties of polyaniline/organic clay nanocomposites doped with dodecylbenzene sulfonic acid and EPDM rubber conductive composites. The conductivity of the nanocomposites is as high as 10^−3^ S·cm^−1^, and they have an excellent mechanical performance, as well as high microwave attenuation values in the range of 8–12 GHz. Therefore, polyaniline is added to the silicone as a wave absorber to prepare polyaniline/silicone coatings, it is important to develop a marine silicone antifouling coating with certain wave absorption for some ships that are serviced above and below the sea.

In this paper, four acidified polyanilines were mixed into polydimethylsiloxane (PDMS) and coatings with both microwave absorbing and antifouling properties were prepared. The chemical structure, microscopic morphology and electromagnetic parameters of polyaniline powder were investigated. The surface and interface properties, mechanical properties, and anti-fouling performance of the coatings were investigated, and then the antifouling mechanism of these coatings was proposed accordingly.

## 2. Experimental Section

### 2.1. Materials

The reagents used in this study are hydroxyl-terminated polydimethylsiloxane (PDMS, Mn = 60,000, 10,000 cp) and methyltriethoxysilane (D150), both purchased from Shandong Dayi Chemical Co., Ltd. (Laiyang, China). Conductive polyaniline doped with hydrochloric acid (PANI-HCl, Mn = 55,000, particle size = 0.5 μm, ρ= 1.291 g/cm^3^), sulfuric-acid-doped conductive polyaniline (PANI-H_2_SO_4_, Mn = 50,000, particle size = 0.5 μm, ρ = 1.313 g/cm^3^), phosphoric-acid-doped conductive polyaniline (PANI-H_3_PO_4_, Mn = 55,000, particle size = 0.5 μm, ρ = 1.414 g/cm^3^), sulfonic-acid-doped conductive polyaniline (PANI-DBSA, Mn = 50,000, particle size = 0.5 μm, ρ = 1.394 g/cm^3^), all purchased from Chongqing Yuanyuanxiang Technology Co., Ltd. (Chongqing, China). Anhydrous ethanol (AR) and xylene (AR) were purchased from Tianjin Jinhuitaiya Chemical Reagents Co., Ltd. (Tianjin, China); cyclohexanone (AR), paraffin, and NEM conductive tape were purchased from Merck Co., Ltd. (Shanghai, China); and acetone (CP) and dibutyltin dilaurate (DBTDL, AR) were purchased from Thermo Fisher Scientific Co., Ltd. (Shanghai, China). Peptone, nutrient agar and yeast extract were all purchased from Beijing Aoboxing Biotechnology Co., Ltd. (Beijing, China); iron phosphate (FePO_4_, CP) was purchased from Jinan Meng Qiao Chemical Co., Ltd. (Jinan, China); magnesium carbonate (MgCO_3_, CP) and ethyl orthosilicate (TEOS, AR) were purchased from Zehui Chemical Co., Ltd. (Wuxi, China). *Navicula* were purchased from the Algae Germplasm Bank of the Chinese Academy of Sciences (Qingdao, China); sea water was taken from the Yellow Sea (Dalian, China).

### 2.2. Preparation of the Coatings

The coating is composed of component A, component B of the curing agent, and component C of catalyst. The specific preparation process is as follows.

Preparation of component A: Add PDMS into BGD7500 grinding dispersion mixer (Guangzhou Biuged Laboratory Instrument Co., Ltd., Guangzhou, China) and adjust the speed to 1200 rpm/min. Add each kind of polyaniline powder according to the 30% mass of polyaniline/PDMS, and make the mixture more uniform with the aid of a paint mixing knife. Then, increase the rotating speed to 4000 rpm/min and disperse for 30 min to ensure that PDMS and polyaniline are evenly mixed. After mixing, put the solution in the tank for 24 h. Component A can then be obtained.

Preparation of component B: prepare mixed solvent (xylene:cyclohexanone:anhydrous ethanol = 7:1:2), take ethyl orthosilicate:mixed solvent = 3:7 to obtain component B of the curing agent.

Preparation of component C: take dibutyltin dilaurate:mixed solvent = 2:5 to prepare component C of the catalyst.

The coatings were prepared by controlling 30 Vol.% PANI to PDMS and the mass ratio of three components is as follows: component A:component B:component C = 20:4:1.

Sample markings: four acid-doped polyanilines—PANI), hydrochloric-acid-doped conductive polyaniline (PANI-HCl)—PH, sulfuric-acid-doped conductive polyaniline (PANI-H_2_SO_4_)—PS, phosphoric-acid-doped conductive polyaniline (PANI-H_3_PO_4_)—PP, and sulfonic-acid-doped conductive polyaniline (PANI-DBSA)—PD, polyaniline and PDMS coating—PANI-P, acid-doped polyaniline/PDMS (PANI-HCl-PDMS)—PH-P, sulfuric acid-doped polyaniline/PDMS (PANI-H_2_SO_4_-PDMS)—PS-P, phosphoric-acid-doped polyaniline/PDMS (PANI-H_3_PO_4_-PDMS)—PP-P, and sulfonic acid-doped polyaniline/organosilicon (PANI-DBSA-PDMS)—PD-P.

### 2.3. Characterization

#### 2.3.1. Sedimentation Rate

Add 10 mg of PANI before or after modification to 15 mL of deionized water, use JY92-IID ultrasonic cell grinder (Ningbo Haishu Wufang Ultrasonic Equipment Co., Ltd., Ningbo, China) to disperse for 5 min, obtain the powder suspension, and leave it in a colorimetric dish for 30 min. Measure the suspension using a UV–visible spectrophotometer, and measure the absorbance value OD600 of the suspension every 3 min to obtain the powder sedimentation rate.

#### 2.3.2. Morphology and Structure

##### Scanning Electron Microscopy (SEM)

Bond the coating upward to the metal block with a conductive adhesive, and spray gold on the sample using a JFC-1100 ION sputter (JEOL Ltd., Tokyo, Japan). Set the current mode to DC, adjust the voltage to 5 kV, adjust the current to 5 mA, and spray gold on the sample for 3 min. The morphology of PANI and the coating was observed using SEM (Supra-55-sapphire, Carl Zeiss AG, Jena, Germany). The observation mode was SE2, and the acceleration voltage was 1 kV.

##### Confocal Laser Scanning Microscope (CLSM)

The morphology of the coatings was observed and analyzed using OLS4000 laser confocal microscope (Olympus, Tokyo, Japan). Surface roughness (Sa) and line roughness (Ra) of the coatings were measured via LEXT analysis software.

##### Fourier Transform Infrared Spectrometry (FTIR)

The FTIR spectrum of PANI was analyzed using the KBr press method. Then, 100 mg of KBr and 1 mg of PANI were mixed and ground in an agate mortar of φ = 50 mm for 2 min, and then placed in a mold with φ = 10 mm, in which the pressure was increased to 25 MPa, and the solution was left for 2 min. The mold was then removed, and the flakes were placed in a flake holder for infrared tests. Fourier transform infrared spectrometry (PerkinElmer, Waltham, MA, USA) was used to scan the samples 32 times, with a scanning range of 450–4000cm^−1^ and resolution of 2 cm^−1^.

#### 2.3.3. Contact Angle and Surface Energy

Using a JC2000C contact angle meter (Shanghai Zhongchen Digital Technology Equipment Co., Ltd., Shanghai, China), the contact angle was measured via the hanging drop method. Next, 2 μL liquid (deionized water or diiodomethane) was dropped onto the sample with a syringe at room temperature, and the contact angle was observed and recorded simultaneously. The surface energy of the coating was calculated via the two-liquid method of Owens [21].

#### 2.3.4. Tensile Test

Referring to the standard GB/T528-2009, the coating is divided into samples by using a cutter 75 mm × 25 mm × 4 mm, using UTM5105 microcomputer controlled electronic universal material testing machine (Jinan Wance Electrical Equipment Co., Ltd., Jinan, China) to stretch at the speed of 25 mm/min to obtain the breaking strength and maximum elongation. The elastic modulus was calculated by selecting the slope stress–strain curve within the strain of less than 0.1 mm/mm. Three parallel measurements were made for each coating.

#### 2.3.5. Conductivity Characterization

The BJ-15 powder tablet press (Tianjin Boxun Technology Co., Ltd., Tianjin, China) was used to press the prepared polyaniline powder into a circular plate with a diameter of 13 mm and a thickness of 1 mm under a pressure of 15 MPa. The specific details are consistent with the above infrared tablet. An SZ82 digital four-probe tester (Suzhou Telecom Instrument Factory, Suzhou, China) was used to measure the resistivity of the samples at room temperature.

#### 2.3.6. Electromagnetic Characteristic

Electromagnetic parameters were tested using the coaxial transmission line method. The paraffin was mixed with 40 wt.% PANI powder and then pressed into a ring (Inner and outer diameter values of 3 mm and 7 mm, and a thickness 3 mm). A TFN FMT800 vector grid analyzer (Shanghai huangyuan Xinke Technology Co., Ltd., Shanghai, China) was used to measure the complex dielectric constant (ε_r_) and the complex permeability (μ_r_) of every ring in the range of 2–18 GHz.

#### 2.3.7. Anti-Fouling Test

(1) Anti-bacteria test

The coating was tested for resistance to the adhesion of marine bacteria. The anti-fouling performance was measured by comparing the bacterial adhesion rate and removal rate on the coating. The solid medium method was used to evaluate the resistance of the coating to bacterial adhesion. A Zobell 2216E medium was used as the colony medium, and the medium was stirred in a water bath at 90 °C until it was completely dissolved, and then sterilized in an autoclave at 0.1 MPa for 20 min. The sterile seawater, beakers, measuring cylinders, cotton swabs and applicators were then autoclaved for 30 min and placed on the clean bench. In addition, all samples (six slides: three for washing and three for rinsing) were completely immersed in fresh seawater for 24 h. Three slides were placed in 50 mL of sterilized seawater for rinsing, and the rest were placed in a 50 mL centrifuge tube containing 35 mL of sterilized seawater. They were then placed in a speed-controlled multipurpose oscillator with 20 mm amplitude of vibration at the speed 150 rpm/min for 15 min. After diluting 10^6^ times, 10 μL of bacterial solution was collected and spread evenly on 2216E solid culture medium (its formula is shown in Table 1). The medium was placed upside down in the biochemical incubator for 48 h at 30 °C, and the bacterial colonies on the medium were then photographed and recorded.

Image Pro Plus software (Version 5.1) was used to count the bacterial cover area on each medium of the rinsed and washed sample. The bacterial removal rate (R) was calculated as the ratio of the difference between the colony concentration of rinsed (C_Rinse_) and washed (C_Wash_) to the percentage of colony concentration of rinsed sample, as shown in Equation (1).

(1) $$R=\frac{\left(C_{Rinse}-C_{Wash}\right)}{C_{Rinse}}\times 100\%$$

(2) Anti-diatom test

The anti-diatom adhesion test was also conducted on the coatings. A *Navicular* benthic diatom was used to evaluate the antifouling effect of the coating. Its *Chlorophyll-a*, which attached on the coating was measured. The detailed operation procedure is as follows:

(1) The filtered seawater was sterilized in an autoclave at 0.1 MPa for 40 min, cooled to room temperature and then removed and set aside.
(2) Six slides of each coating were immersed in *Navicula* suspension and incubated in a light incubator for 24 h at 22 °C to control the light ratio of continuous light:no light = 12 h:12 h. Three slides were gently rinsed with sterilized seawater, and the other three slides were placed in a 50 mL centrifuge tube containing 35 mL of sterilized seawater and shook on a speed-controlled multipurpose oscillator with 20 mm amplitude of vibration at the speed 150 rpm/min for 15 min.
(3) Each slide was placed into a test tube with a concentration of 90% acetone solution, 45 mL per test tube. Then, 1 mL of 1% magnesium carbonate suspension was added dropwise, and the test tubes were sealed and put into a dark environment at 4 °C for 24 h to extract chlorophyll.
(4) After extraction, 10 mL of the supernatant was placed into a centrifuge tube and centrifuged at 4000 rpm/min for 15 min. Next, 3 mL of the supernatant was dropped into a quartz cuvette and absorbance values at 630, 645, 663 and 750 nm were measured using a UV spectrophotometer, and *Chlorophyll-a* value (*ρ_a_*) was calculated via Equation (2).

(2) $$\rho_{a}=11.64\times 2.16\left(OD663\right)-2.16\times \left(OD645\right)+0.10\times (OD630)$$

## 3. Results and Discussion

### 3.1. Properties of PANI Powder

#### 3.1.1. Sedimentation Rate of PANI Powder in Water

**Figure 1:**
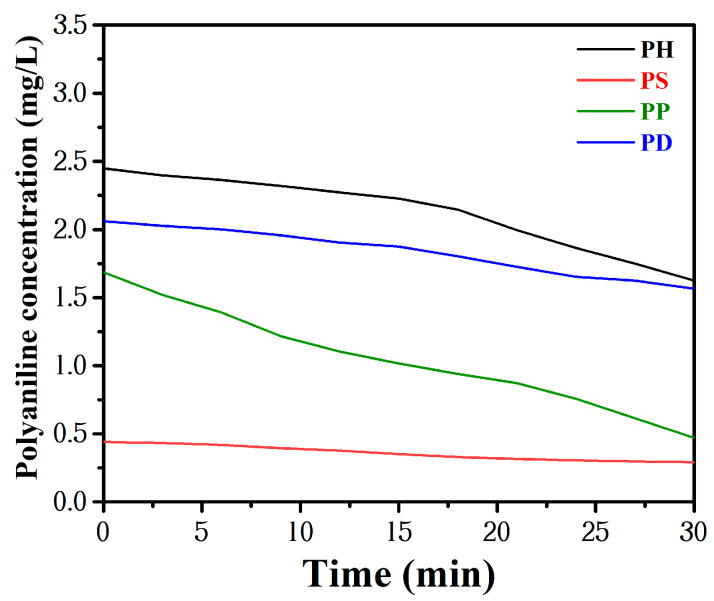
Sedimentation rate curves of the studied PANI powder.

PANI powder has small particle size and size effect, surface effect and tunneling effect. However, it also has the disadvantages of large specific surface energy and easy agglomeration, and this agglomeration will also cause it to lose some of its original effects and affect the properties of the material itself [21,22]. The concentration of the PANI suspension was measured every 3 min using a UV–Vis spectrophotometer, and the settling rates of different PANI were compared via the settling rate curves, where the wavelength used for testing the settling rate was 600 nm, as shown in Figure 1. All PANI powder significantly precipitated after standing for some time. The initial concentration of PS was only 0.44 mg/L after precipitation, and PP settled rapidly, falling from 1.68 mg/L to 0.45 mg/L within 30 min, while PH showed a more stable sedimentation trend in deionized water.

#### 3.1.2. Morphology and Structure of PANI Powder

**Figure 2:**
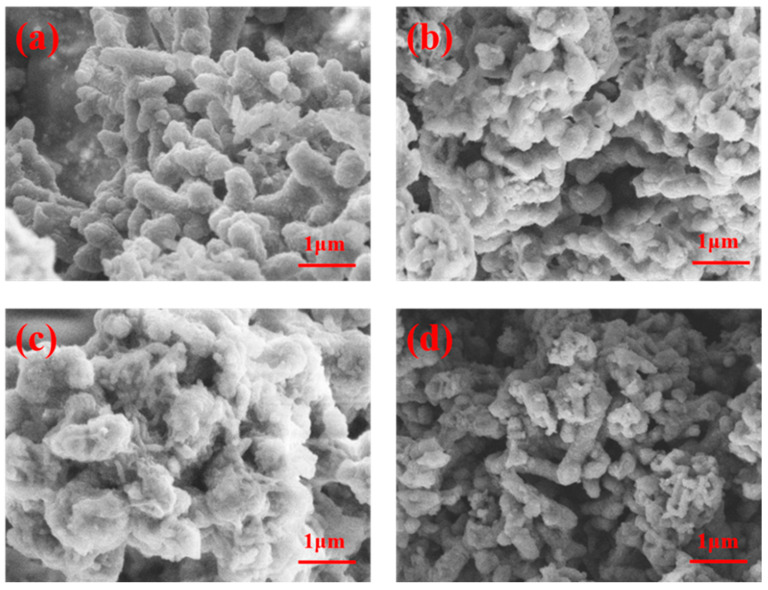
SEM morphology of PANI powder: (**a**) PH; (**b**) PS; (**c**) PP; (**d**) PD.

The morphology of PANI powders was observed by SEM, and it can be seen that the PH, PS, and PD powders are short and rod-like, as shown in Figure 2a,b,d, where the diameter and length values of the PH short rod mount were more prominent and regular, while the phosphoric-acid-doped polyaniline (Figure 2c) agglomerated into a lump.

**Figure 3:**
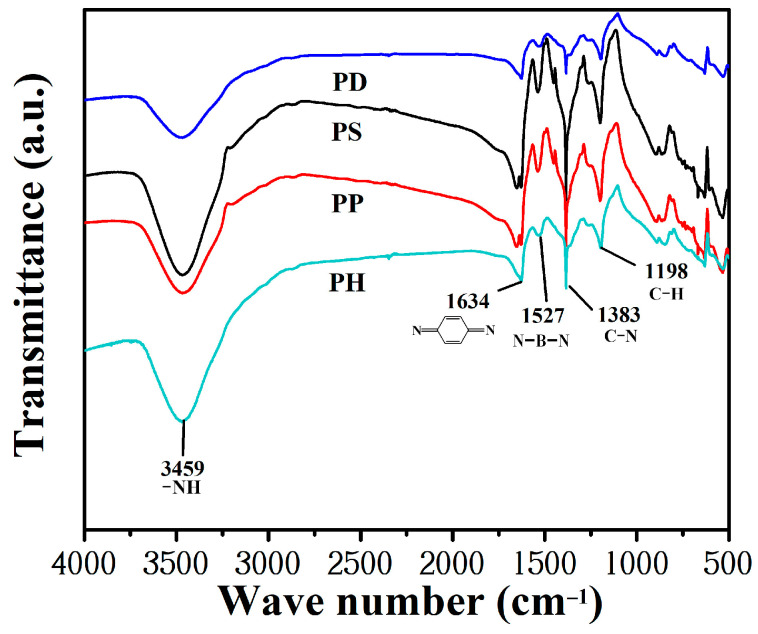
FTIR spectrum of the studied PANI powder.

The FTIR spectrum of four PANI powders are shown in Figure 3. The main characteristic absorption peaks of polyaniline with hydrochloric-acid-doped polyaniline are as follows: 3459 cm^−1^ for the N-H stretching vibration peak of polyaniline, 1634 cm^−1^ and 1527 cm^−1^ for the quinone structure and the benzene ring structure of polyaniline, 1383 cm^−1^ corresponding to the C-N absorption vibration peak, and 835 cm^−1^ and 1198 cm^−1^ for the out-of-plane and in-plane C-H bending vibration peaks of the benzene ring, the intensity of which represents the degree of PANI doping. The absorption peaks of the quinone ring, benzene ring and amine group were present for each doped polyaniline in the FTIR spectra.

#### 3.1.3. Conductivity of PANI Powder

The conductivity values of different PANI powders are shown in Table 2. Clearly, the conductivity values of polyaniline doped with different substances are quite different. The conductivity of PD is the lowest: only 0.85 mS·cm^−1^. That of PP is largest (10.8 mS·cm^−1^); it is more than 12 times that of PD. This phenomenon indicates that the acidity has a great influence conductivity. Generally, polyaniline doped with stronger acids has a higher conductivity, weaker acids such as organic acids cause polyaniline to have a lower conductivity, and smaller-sized acid chains can diffuse into polyaniline chains more rapidly, which is a more effective doping process compared to long chains. The conductivity of aniline derivatives substituted on the quinone ring imine group is related to the length of the alkyl substituent, with longer substituents decreasing the conductivity [23], thus explaining the higher conductivity of inorganic acid-doped polyaniline compared to organic acid-doped polyaniline and the lower conductivity of the salts [24]. The higher conductivity of phosphoric-acid-doped polyaniline is caused by the presence of side chain hydroxyl groups in the phosphoric acid molecule, which has a conjugation effect of providing electrons and enhancing the conjugation effect on the main chain of polyaniline, leading to the enhancement of the conductivity of the product [25].

#### 3.1.4. Electromagnetic Parameters of PANI Powder

**Figure 4:**
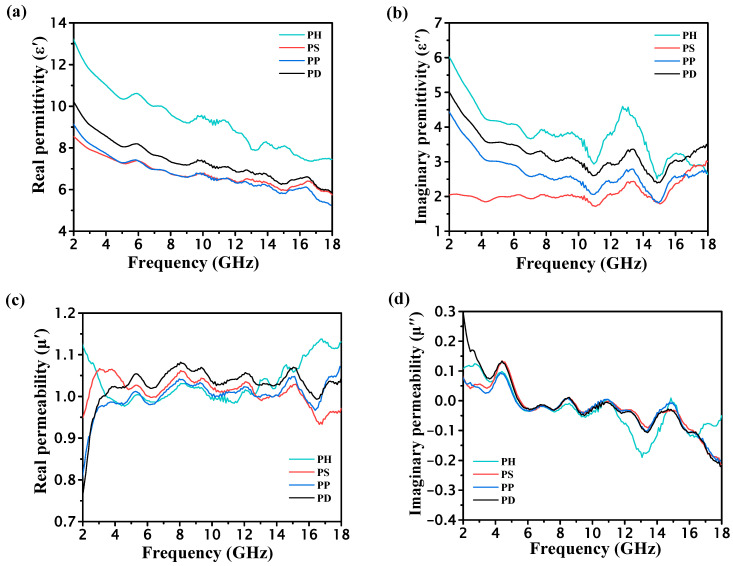
Studied PANI powders: (**a**) ε′; (**b**) ε″; (**c**) μ′; (**d**) μ″.

The real part (ε′) and imaginary part (ε″) of the complex permittivity and the real part (μ′) and imaginary part (μ″) of the complex permeability of several groups of materials were tested separately. The related results are shown in Figure 4.

It can be seen that the dielectric constant and permeability constant of polyaniline doped with different acids is different. The directional polarization and relaxation polarization of PANI under the action of microwave electric field cause dielectric loss, which mainly derives from four factors. (1) The first is resistance loss, where the various polarization mechanisms of a medium compete with changes in the electric field when the polarization loss is weak [26]. (2) The second is fetching polarization and chirality polarization loss, when the applied electric field frequency increases to a certain value, polarization cannot compete with the changes in the electric field, and the movement of charged masses (such as polaritons) absorbs the external electric field energy, so that the electromagnetic energy is transformed into thermal vibrational energy. (3) The third is interface polarization loss, including quantum loss and orientation polarization loss caused by the interface. (4) The fourth is both dielectric structure loss and resonance loss. Structure loss is mainly caused by the irregular structure of the conductive material, or by the loss caused by containing the second phase. Resonance loss is mainly caused by the natural resonance generated by the applied electromagnetic field, including diffusion resonance loss, size resonance loss, etc.

With the increase in frequency, both the real and imaginary parts of the complex permittivity of each PANI powder show a decreasing trend. The imaginary parts of the permittivity fluctuate in a high frequency range, and the real and imaginary parts of the complex permeability also show a more consistent trend. In addition, PH exhibits the strongest complex permittivity in both real and imaginary parts among the four materials. Microwave absorption performance is determined by its microwave loss characteristics and impedance matching holding together. Too high a conductivity and a mismatched dielectric/dielectric response often affect impedance matching characteristics and make it impossible to achieve better microwave absorption.

**Figure 5:**
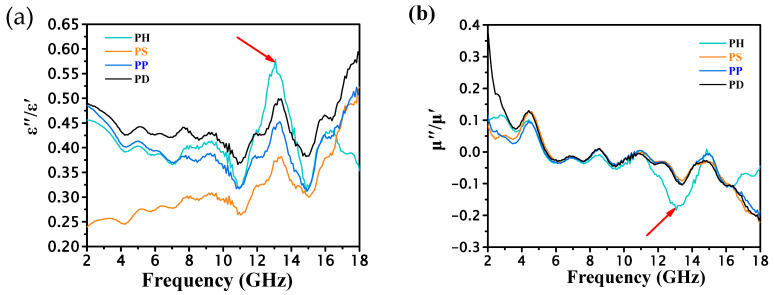
Studied PANI powder: (**a**) ε″/ε′; (**b**) μ″/μ′.

Generally, the microwave loss characteristics of PANI can be evaluated via the dielectric loss factor, tan(ε″/ε′), and magnetic loss factor, tan(μ″/μ′), as shown in Figure 5. Clearly, the dielectric losses of PH at 2–18 GHz are significantly stronger than those of the other materials. More importantly, when comparing the dielectric loss factor and magnetic loss factor, it can be found that there is one highly matched dielectric/dielectric response peak in the mid-frequency range, which is located at around 13 GHz (marked by the red arrow in Figure 5). The dielectric/demagnetization losses in the intermediate frequencies mainly originate from the polarization relaxation process in the dielectric. The high match between dielectric and dielectric response indicates that the special structural units in the doped polyaniline material are capable of polarization relaxation in response to both electric and magnetic field changes. The analysis suggests that this is caused by the special polarization structure in the doped material.

By comparison, it can be seen that PH not only has stronger dielectric loss characteristics, but also stronger dielectric loss characteristics matched with it, i.e., it satisfies both strong microwave loss characteristics and good impedance matching characteristics [27]. On the one hand, polarization can effectively regulate the conductivity of PANI, thus better achieving the impedance matching of the material itself. On the other hand, the special polarization system not only induces the corresponding dielectric polarization relaxation, but also causes matching dielectric polarization relaxation, and further realizes the conjugate enhancement of dielectric and dielectric magnetism.

#### 3.1.5. Electromagnetic Properties of PANI Powder

**Figure 6:**
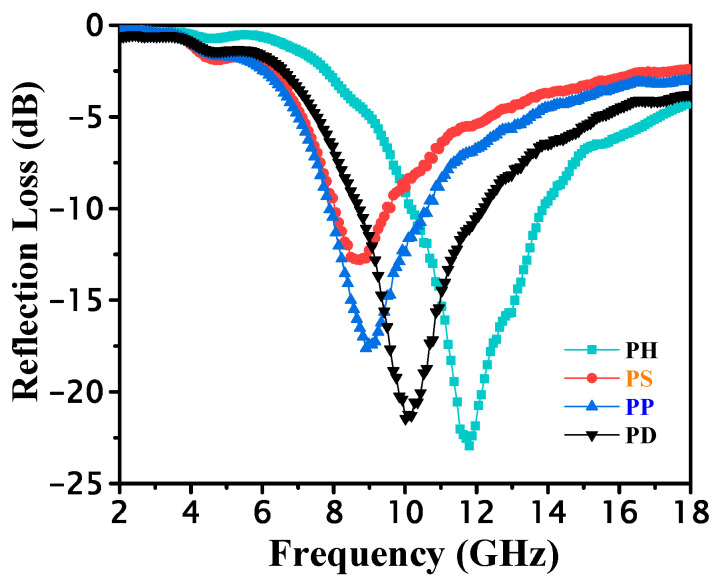
Calculated RL at 2–18 GHz for the studied PANI with thickness of 3.1 mm.

Based on the complex permittivity (*ε_r_*) and complex permeability (*μ_r_*), of the sample obtained in the test, the theoretical simulation of the microwave reflection loss (*RL*) of the material can be calculated via Equation (3), where f is the frequency of the electromagnetic microwave, c is the speed of light under vacuum condition, and d is the thickness of the material. *μ_r_* and *ε_r_* are the measured relative complex permittivity and magnetic permeability values of the material, respectively. The *RL* results calculated using the above method at a thickness of 3.1 mm are shown in Figure 6.

(3) $$RL=20\mathrm{lg}\left|\frac{\sqrt{\mu_{r}/\varepsilon_{r}\;}\tanh\left[\left(j2\pi \mathrm{fd}/c\right)\sqrt{\mu_{r}/\varepsilon_{r}}-1\right]}{\sqrt{\mu_{r}/\varepsilon_{r}}\tanh\left[\left(j2\pi \mathrm{fd}/c\right)\sqrt{\mu_{r}/\varepsilon_{r}}+1\right]}\right|$$

Comparing the RL values of different conductive polyaniline at a thickness of 3.1 mm, it can be seen that all acid-doped polyanilines have microwave absorption capability, and the range of RL ≤ −10 dB (microwave loss over 90%) is essentially concentrated in the middle and high-frequency bands, and the peak reflection loss is optimal in the range of 2–18 GHz for PH, as shown in Table 3. The peak loss of PH at 11.77 GHz reaches −23.15 dB, and the bandwidth of RL curve below −10 dB (effective bandwidth) can reach 3.81 GHz (10.11–13.92 GHz), which shows excellent absorption performance. The overall electromagnetic loss is consistent with the above electromagnetic parameter analysis, which once again shows that four doped polyanilines are mainly based on dielectric loss, and the applied electromagnetic field acts on the polyaniline. The dipole will adjust its own direction so that it is parallel with the electromagnetic field direction, causing the polarization of the polarizer, and the process of adjusting the direction needs to overcome the electromagnetic field effect, which causes energy loss.

### 3.2. Properties of the Coatings

#### 3.2.1. Tensile Properties

**Figure 7:**
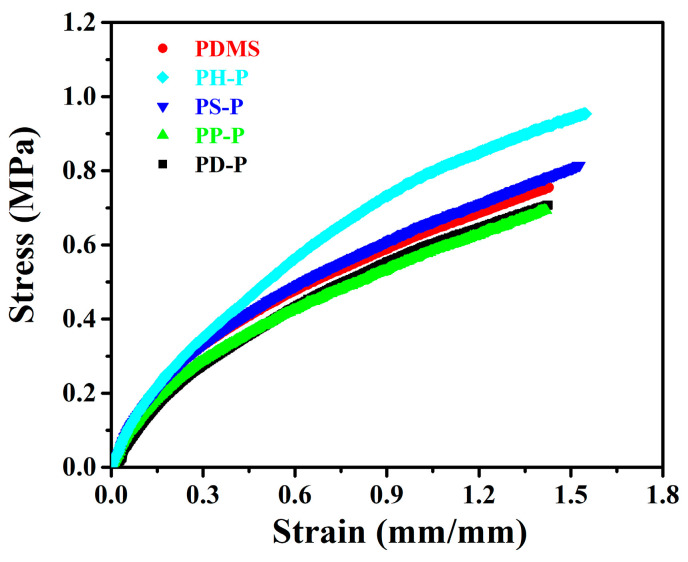
Stress–strain curves of the coatings.

The stress–strain curves of the coating are shown in Figure 7. The tensile properties of PDMS specimens were higher than those of PP-P and PD-P, with a breaking strength of 0.76 MPa and an elongation of 143%, because the silicone elastomer formed a dense mesh structure after cross-linking and curing, which enabled it to have good mechanical properties. Meanwhile, PH-P and PS-P have stronger mechanical properties than PDMS, with breaking strength values of 0.95 MPa and 0.81 MPa and an elongation of 155% and 153%, respectively, because polyaniline was dispersed in the silicone matrix. This is because when the coating is deformed by external forces, the polyaniline particles can absorb the external forces and reduce the external forces on the silicone. Therefore, the addition of PH and PS enhances the interaction force between the polyaniline particles and the substrate interface, thus improving the mechanical properties of the coating. The difference of elastic modulus of studied coatings is small, as shown in Table 4.

#### 3.2.2. Morphology of the Coatings

**Figure 8:**
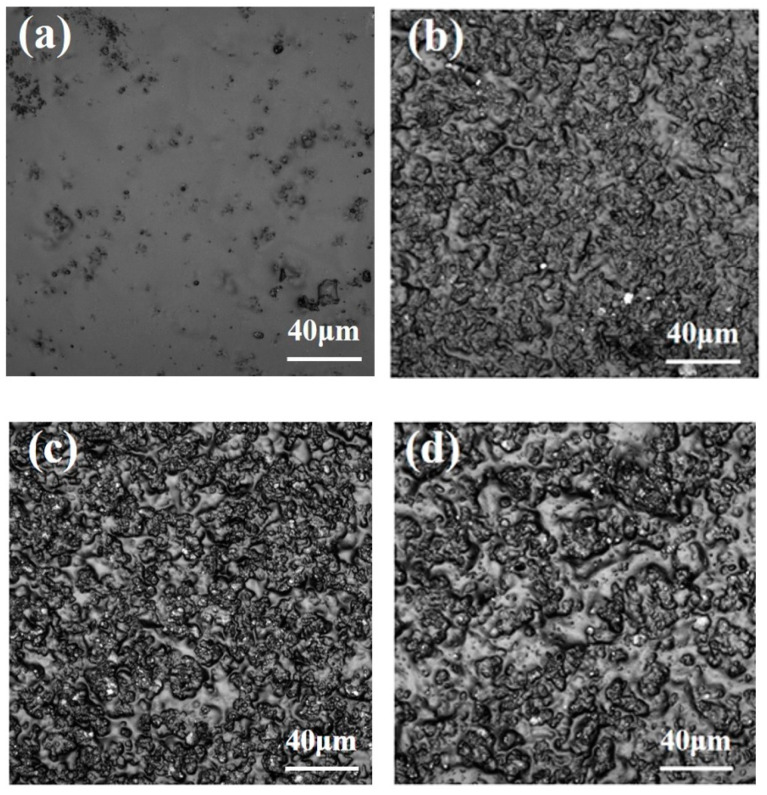
CLSM morphology of the coatings: (**a**) PH-P; (**b**) PS-P; (**c**) PP-P; (**d**) PD-P.

**Figure 9:**
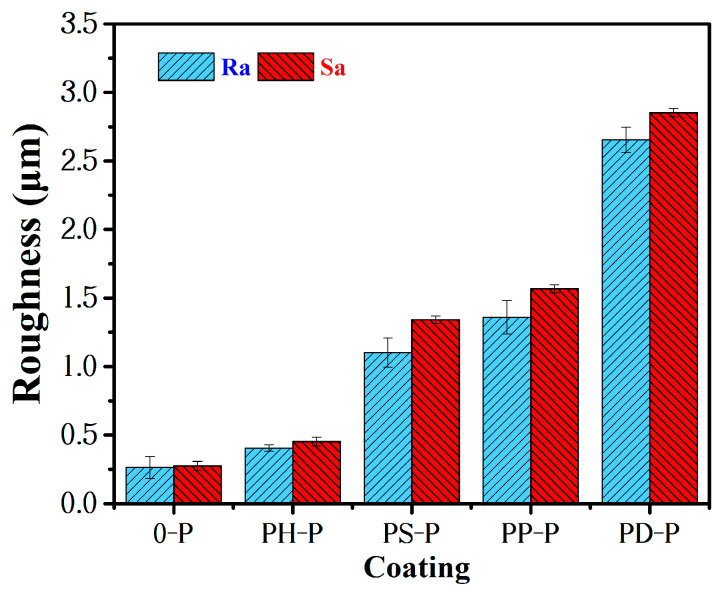
The roughness of the coatings.

The CLSM morphology of the coatings are shown in Figure 8, and their roughness is shown in Figure 9. For the specimens without adding polyaniline, the Ra and Sa of PDMS coating were lowest, while the roughness of other specimens increased due to additional PANI. This is because the special structure of polyaniline and its composites tend to create the micro- and nano-graded structure of the coating, which is easy to form small agglomerates for some areas that are not completely dispersed, resulting in the unevenness of the coating and an increase in roughness.

#### 3.2.3. Surface and Interface Characteristics

The measured contact angles and surface energies of the coatings are shown in Table 5. The average water contact angle of the coatings is more than 100°, showing good hydrophobicity. Combined with the surface energy data, it is evident that PH-P has a lower surface energy compared to PDMS: only 17.17 mJ/m^2^. Additionally, the shape of water droplets may be affected by the roughness value, mainly because silicone is a hydrophobic coating. After adding polyaniline, the micro-nano structure of its surface will cause the water contact angle to become larger: the roughness is PH-P < PS-P < PP-P < PD-P. The water contact angles can be observed in PS-P < PP-P < PD-P, but the water contact angle of the PH-P specimen is the largest. There is uneven distribution of powder in some areas measuring contact angle [28].

#### 3.2.4. Antifouling Performance of the Coatings

##### Adhesion Resistance to Marine Bacteria

**Figure 10:**
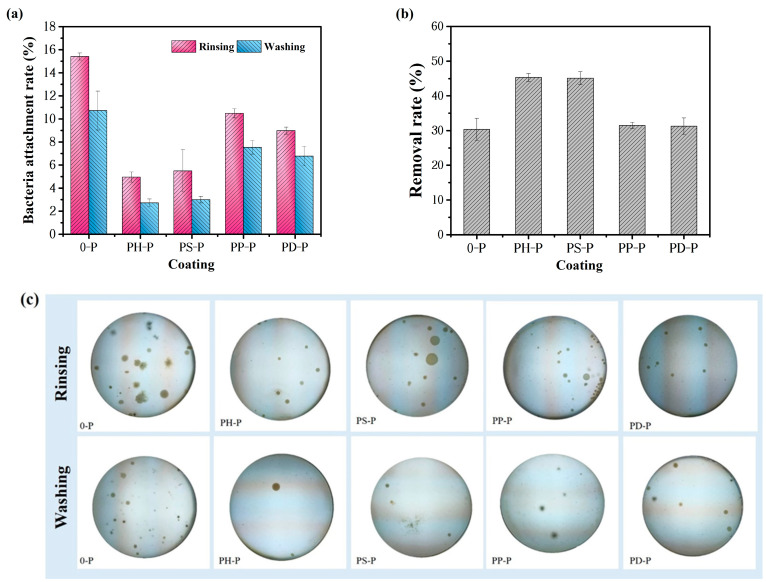
Anti-bacteria performance of the coatings: (**a**) bacterial adhesion rate; (**b**) removal rate of adhered bacteria; (**c**) bacterial colony photos on the medium at 72 h.

The resistance of the coatings to marine bacterial adhesion was evaluated using a solid media method. Both rinsing and flushing were used for the treatment, and the bacterial adhesion rates were calculated, as shown in Figure 10a. The coating added without polyaniline had an average performance against bacterial biofilm adhesion, while the other coatings added to PANI performed well in the test, where the bacterial adhesion rates of PH-P and PS-P specimens were 4.95% and 5.49% after rinsing treatments, respectively, and were reduced to 2.72% and 3.01% after flushing treatments. Bacteria form a basement film on the coating, which is formed by the physical adsorption of some organic molecules of bacteria (proteins, polysaccharides, and glycoproteins) to the surface of the substrate within a few seconds and enrichment to a certain extent to form the basement film, which is then formed on the conditioned film. Therefore, based on the interface properties of the PANI coating, it is clear that the synergistic effect of both low surface energy and hydrophobicity will make it difficult for the bacteria to form the basement film, and their physisorption will become weak at the basement film stage.

The calculated removal rates of adhered bacteria on the coatings are shown in Figure 10b, and adding polyaniline could effectively improve the bacterial removal rate of the coatings. Without the addition of polyaniline, the removal rate is only 30.37%, while those of PH-P and PS-P coatings are 45.35% and 45.14%, respectively, which is significantly greater than that of the other two materials.

##### Anti-*Navicula* Attachment Behavior and Antifouling Mechanism

**Figure 11:**
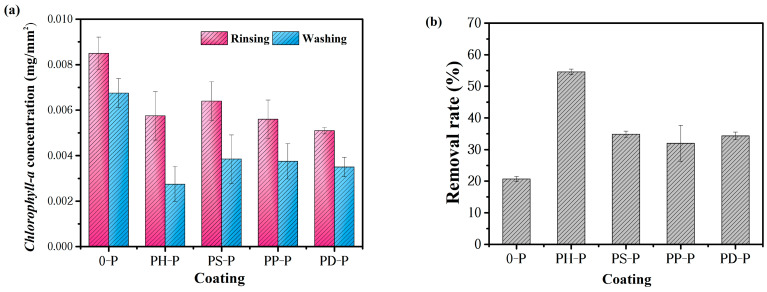
Anti-*Navicula* adhesion tests: (**a**) adhesion rate with rinsing and washing; (**b**) Removal rate of *Navicula* attached on the coatings.

Pure PDMS effectively achieves antifouling due to its low surface energy and hydrophobicity. Combining PANI with PDMS, as shown in Figure 11, the *Chlorophyll-a* values obtained by PH-P rinsing and flushing are lower: 0.0057 mg/L and 0.0028 mg/L, respectively. The desorption rate is even higher than 54.62%, indicating that the amount of *Navicula* attached to it is low, compared with PDMS. Adding a certain amount of polyaniline coating has better antifouling properties than PDMS, i.e., the antifouling properties of both of them play a synergistic role; this is consistent with above anti-bacterial adhesion tests.

Combined with research on the anti-stick mechanism of polyaniline [5], in this paper, the following hypotheses were proposed for the antifouling mechanism of polyaniline materials. The adhesion of fouling organisms, whether microbial membranes or large fouling organisms, is associated with small molecules of DOPA (3,4-dihydroxyphenylalanine, dopa). The molecular chain structure of polyaniline has three main structures: intrinsic state, unipolarons, and dipolarons. The charge distribution of the molecular chain structure of polyaniline was previously calculated in the literature [29] via quantum mechanics. It was concluded that N atoms at the ends of the quinone ring in the intrinsic state polyaniline molecular chain are positively charged, and C atoms on the benzene ring are more negatively charged. DOPA adhesion molecules are mainly adsorbed with the active center of -OH in the adhesion process, and these two groups have a certain electron-absorbing effect.

The *Navicula* test results show that the anti-benthic diatom effect of inorganic acid-doped polyaniline coating was greater than that of the organic acid-doped polyaniline coating, likely because the inorganic acid-doped polyaniline was more stable than the organic acid-doped polyaniline. The solubility of inorganic acid-doped polyaniline in the solvent was larger, and the inhibitory effect on algae was better reflected; therefore, the anti-benthic diatom performance of inorganic acid-doped polyaniline was greater [30].

#### 3.2.5. Electromagnetic Absorption Properties of the Coatings

**Figure 12:**
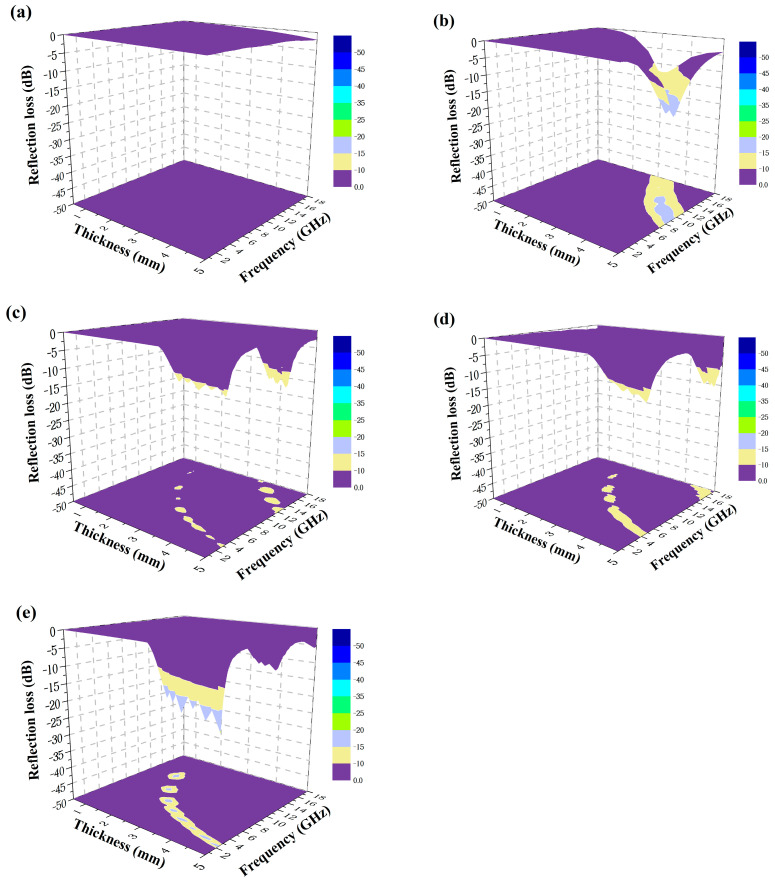
3D microwave absorption effect of the coatings (absorbing layer thickness 0–5 mm). (**a**) 0-P; (**b**) PH-P; (**c**) PS-P; (**d**) PP-P; (**e**) PD-P.

The RL values of the coatings were calculated at different thicknesses (0–5 mm) and the 3D absorbing effects of the coatings were plotted, as shown in Figure 12. Within the range of 0–5 mm thickness, the 0-P coating essentially has no absorption because PDMS is a microwave transmitting material, and the coating containing PANI has a strong microwave loss intensity and significantly increased microwave loss bandwidth. The peak of RL reached −19.61 dB at 6.6 GHz, while the peaks of RL of the coating were only −10 dB and −17.5 dB in the 8–12 GHz range when the polyaniline additions were 24 wt.% and 30 wt.% in the ternary co-blended coating studied by Faez et al. [19]. By regulating the thickness of the absorbing layer, the coating is able to achieve more than 90% loss of microwave in the range of 6–18 GHz. It is worth mentioning that the microwave loss performance of PANI coating is slightly reduced compared with that of pure PANI powder, which is because the electromagnetic microwave starts to be consumed after entering the coating. As the ratio of polyaniline gradually increases from the surface layer to the bottom layer, the surface layer of powder is more sparsely dispersed, and the bottom layer is denser. When electromagnetic microwave enters the coating, they are consumed layer by layer, reducing the reflection of electromagnetic microwaves.

### 3.3. Discussion

#### 3.3.1. Relationship between Bacterial Removal Rate and Relative Bonding Force of the Coating

Brady et al. [31] proposed that the relative bonding force (*A_r_*) between the fouling organisms and the coating is proportional to the 1/2 power of the product from the elastic modulus (*E*) and the surface free energy (*σ_s_*) of the coating, as shown in Equation (4).

(4) $$A_{r}\propto \;{\left(E\sigma_{S}\right)}^{\frac{1}{2}}$$

**Figure 13:**
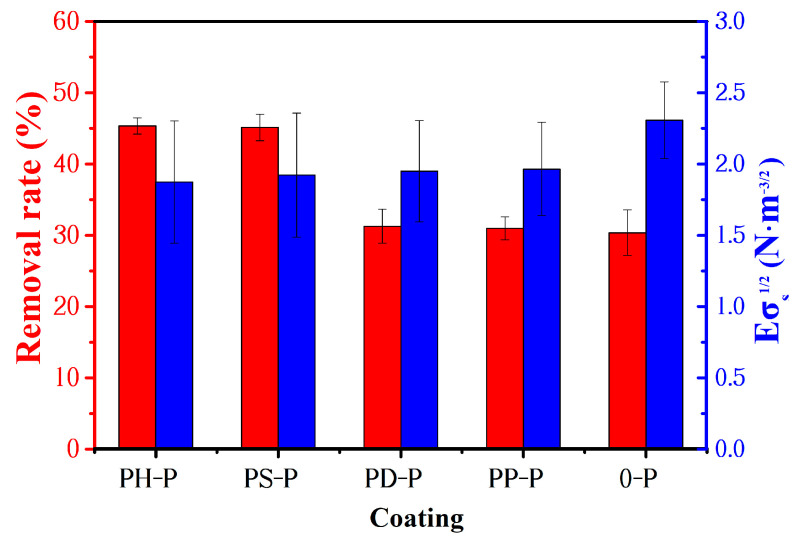
The relative bonding force and bacterial removal rate of the coatings.

Relative bonding force is considered to be one of the important factors for designing and screening silicone low-surface-energy antifouling coatings, i.e., reducing the elastic modulus and surface energy of the coating can lead to a lower adhesion of fouling organisms and improved antifouling performance. The experimental results in this paper are consistent with this concept. The anti-benthic diatom and anti-marine bacterial adhesion tests revealed a consistent trend regarding the change in the removal rate with increasing PANI content, so the removal rate of adhered bacteria on the PANI-P coating and its relative bonding force were shown in Figure 13. The order of the magnitude of the relative bonding force between the fouling organisms and the coating was 0-P > PP-P > PD-P > PS-P > PH-P, while the order of the magnitude for the bacterial detachment rate was PH-P > PS-P > PD-P > PP-P > 0-P, which is essentially in accordance with the law proposed by Brady et al. It indicates that PANI has a positive effect on enhancing the antifouling performance of the coating.

#### 3.3.2. Relationship between Bacterial Removal Rate and Roughness

**Figure 14:**
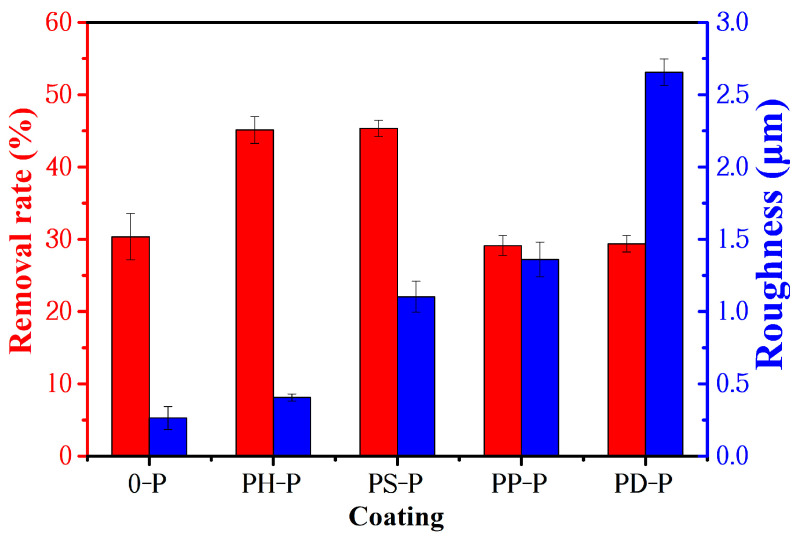
The bacterial removal rate and roughness of the coatings.

The effect of the roughness of the coating on the antifouling efficiency is shown in Figure 14, the surface roughness of the coating increased with the addition of polyaniline powder. The roughness of coating 0-P is 0.264 μm. The minimum roughness of the coating containing polyaniline is 0.406 μm and the maximum roughness is 2.655 μm. For the surface of hydrophobic coating, the increase in roughness is helpful to increase the water contact angle and thus reduce the surface energy of the coating. However, it was also found that too much surface roughness led to an increase in the contact area between the coating and marine bacteria as well as biological secretions, while too high a surface roughness also supports the attachment of bacteria organisms, etc., enabling fouling sea creatures to firmly adhere to the coating. Therefore, the increasing roughness of the coating is not entirely beneficial. In addition, although the roughness of the coating affects the bacteria removal rate of the coating, it is not the main factor. Sufficient roughness can improve the antifouling effect of coatings with a low surface energy.

## 4. Conclusions

(1) The addition of PANI can reduce the surface energy of a coating. Among the studied coatings, the surface energy of coating PH-P is the lowest: only 17.17 mJ/m^2^.
(2) The addition of PANI improves the roughness and mechanical properties of the coating. Coating PH-P has the best mechanical properties, with a maximum fracture strength of 0.95 MPa and a maximum elongation of 155%.
(3) The coating with the addition of PANI showed an improvement in stain resistance. In the bacterial adhesion test, the bacterial removal rate of PDMS sample is only 30.37%. The bacterial adhesion rate of the PH-P coating was 4.95% and 2.72% after rinsing and washing, respectively, and the bacterial removal rate was 45.35%. In addition, in the benthic diatom adhesion test, the chlorophyll values of PH-P coating was 0.0057 mg/L and 0.0028 mg/L after rinsing and washing, respectively, and the removal rate was as high as 54.62%.
(4) Appropriate PANI can enhance the reflection loss of the coating. Coating PH-P reaches the lowest point of RL curve at 11.77 GHz with −23.15 dB, it also shows excellent microwave loss in the range of 2–18 GHz, and the reflection loss reaches −19.61 dB at 6.6 GHz.
(5) Coating PH-P has not only the best antifouling performance, but also lowest surface free energy, which can improve the microwave absorption performance of PDMS, and thus it can be used as an effective antifouling and absorbing coating.

## Figures and Tables

**Table 1:** Formulation of 2216E solid medium.

Composition	Peptone	Yeast Paste	FePO_4_	Nutrient Agar	Seawater
Content	2 g	0.4 g	0.004 g	8 g	400 mL

**Table 2:** The conductivities of the studied PANI powder.

Sample Name	PH	PS	PP	PD
Conductivity (mS·cm^−1^)	1.70	1.69	10.8	0.85

**Table 3:** RL ≤ −10 dB bandwidth and peak of the reflection loss of studied PANI.

Sample	PH	PS	PP	PD
RL ≤ −10 dB Bandwidth (GHz)	3.81	1.55	2.83	3.52
Peak of reflection loss (dB)	−23.15	−12.88	−17.52	−21.52

**Table 4:** Elastic modulus of the coatings.

Sample	PDMS	PH-P	PS-P	PP-P	PD-P
Elastic modulus (MPa)	0.197 ± 0.143	0.204 ± 0.278	0.179 ± 0.116	0.163 ± 0.135	0.161 ± 0.142

**Table 5:** Contact angle and surface energy of the coatings.

Sample	Water (°)	Diiodomethane (°)	Surface Energy (mJ/m^2^)
PDMS	109.05 ± 0.23	63.02 ± 0.34	27.21 ± 0.47
PH-P	117.50 ± 0.52	80.65 ± 0.54	17.17 ± 0.26
PS-P	103.02 ± 0.55	72.30 ± 0.26	20.43 ± 0.30
PP-P	106.32 ± 0.19	73.63 ± 0.67	22.98 ± 0.38
PD-P	110.26 ± 0.64	71.39 ± 0.17	23.62 ± 0.40

## Data Availability

All data in this paper.

## References

[1] DeBerry D.W. (1985). Modification of the electrochemical and corrosion behavior of stainless steels with an electroactive coating. J. Electrochem. Soc..

[2] Mengoli G., Musiani M.M., Pelli B., Vecchi E. (1983). Anodic synthesis of sulfur-bridged polyaniline coatings onto Fe sheets. J. Appl. Polym. Sci..

[3] Tansuǧ G., Tüken T., Özyilmaz A.T., Erbil M., Yazici B. (2007). Mild steel protection with epoxy top coated polypyrrole and polyaniline in 3.5% NaCl. Curr. Appl. Phys..

[4] Armelin E., Oliver R., Liesa F., Iribarren J.I., Estrany F., Alemán C. (2007). Marine paint fomulations: Conducting polymers as anticorrosive additives. Prog. Org. Coatings.

[5] Armelin E., Ocampo C., Liesa F., Iribarren J.I., Ramis X., Alemán C. (2007). Study of Epoxy and Alkyd coatings modified with emeraldine base form of polyaniline. Prog. Org. Coatings.

[6] Xing C., Zhang Z., Yu L., Zhang L., Bowmaker G.A. (2014). Electrochemical corrosion behavior of carbon steel coated by polyaniline copolymers micro/nanostructures. RSC Adv..

[7] Li W., Zhu M., Zhang Q., Chen D. (2006). Expanded conformation of macromolecular chain in polyaniline with one-dimensional nanostructure prepared by interfacial polymerization. Appl. Phys. Lett..

[8] Sathiyanarayanan S., Muthkrishnan S., Venkatachari G. (2006). Corrosion protection of steel by polyaniline blended coating. Electrochim. Acta.

[9] Shahhosseini L., Nateghi M.R., Kazemipour M., Zarandi M.B. (2015). Electrochemical Synthesis of Polymer Based on 4-(2-Furyl) Benzenamine: Electrochemical Properties, Characterization and Applications. Prog. Org. Coatings.

[10] Shahhosseini L., Nateghi M.R., Kazemipour M., Zarandi M.B. (2016). Corrosion protective properties of poly (4-(2-Thienyl) benzenamine) coating doped by dodecyl benzene sulphonate. Synth. Met..

[11] Bandeira R.M., van Drunen J., Tremiliosi-Filho G., dos Santos J.R., de Matos J.M.E. (2017). Polyaniline/Polyvinyl Chloride Blended Coatings for the Corrosion Protection of Carbon Steel. Prog. Org. Coatings.

[12] Wang X.H., Li J., Zhang J.Y., Sun Z.C., Yu L., Jing X.B., Wang F.S., Sun Z.X., Ye Z.J. (1999). Polyaniline as marine antifouling and corrosion-prevention agent. Synth. Met..

[13] Baldissera A.F., De Miranda K.L., Bressy C., Martin C., Margaillan A., Ferreira C.A. (2015). Using conducting polymers as active agents for marine antifouling paints. Mater. Res..

[14] Hou J., Liu S., Jiang X., Waterhouse G.I.N., Zhang Z.M., Yu L.M. (2021). Polyaniline/graphite carbon nitride composite coatings with outstanding photo-induced anodic antifouling and antibacterial properties under visible light. Prog. Org. Coatings.

[15] Yang Z., Peng H., Wang W., Liu T. (2010). Crystallization behavior of poly(ε-caprolactone)/layered double hydroxide nanocomposites. J. Appl. Polym. Sci..

[16] Tiitu M., Talo A., Forsén O., Ikkala O. (2005). Aminic epoxy resin hardeners as reactive solvents for conjugated polymers: Polyaniline base/epoxy composites for anticorrosion coatings. Polymer.

[17] Jamari S.K.M., Kasi R., Ismail L., Nor N.A.M., Subramanian R.R., Ramesh Subramaniam T., Balakrishnan V., Arof A.K.M. (2016). Studies on anticorrosion properties of polyaniline-TiO_2_ blended with acrylic-silicone coating using electrochemical impedance spectroscopy. Pigment Resin Technol..

[18] Cheng B., Wang J., Zhang F., Qi S. (2018). Preparation of silver/carbon fiber/polyaniline microwave absorption composite and its application in epoxy resin. Polym. Bull..

[19] Faez R., Reis A.D., Soto-Oviedo M.A., Rezende M.C., De Paoli M.A. (2005). Microwave absorbing coatings based on a blend of nitrile rubber, EPDM rubber and polyaniline. Polym. Bull..

[20] Zhang C., Liu H., Ju P., Zhu L., Li W. (2018). Effects of pH on the Nickel coating microstructure and internal stress from an additive-free watts-type bath with phytic acid. J. Electrochem. Soc..

[21] Owens D.K., Wendt R.C. (1969). Estimation of the surface free energy of polymers. J. Appl. Polym. Sci..

[22] Blanchini F., Sznaier M. (1995). Persistent disturbance rejection via static-state feedback. IEEE Trans. Automat. Contr..

[23] Seger J., Jarmar T., Zhang Z.B., Radamson H.H., Ericson F., Smith U., Zhang S.L. (2004). Morphological instability of NiSi1-UGeu on single-crystal and polycrystalline Si1-XGex. J. Appl. Phys..

[24] Yu B., Li B. (2006). Fractal-like tree networks reducing the thermal conductivity. Phys. Rev. E.

[25] Wu X., Qian X., An X. (2013). Flame retardancy of polyaniline-deposited paper composites prepared via in situ polymerization. Carbohydr. Polym..

[26] Cheng X., Yokozeki T., Wu L., Koyanagi J., Wang H., Sun Q. (2018). The enhancement effect of carbon-based nano-fillers/polyaniline hybrids on the through-thickness electric conductivity of carbon fiber reinforced polymer. Compos. Part A Appl. Sci. Manuf..

[27] Wang G., Peng X., Yu L., Wan G., Lin S., Qin Y. (2015). Enhanced microwave absorption of ZnO coated with Ni nanoparticles produced by atomic layer deposition. J. Mater. Chem. A.

[28] Gu D.Q., Zhou Y. (2011). An approach to the capsule endoscopic robot with active drive motion. J. Zhejiang Univ. Sci. A.

[29] Li Z., Wang X., Bai H., Cao M. (2023). Advances in Bioinspired Superhydrophobic Surfaces Made from Silicones: Fabrication and Application. Polymers.

[30] Gao M., Quan X., Wang J., Wang Z. (2020). Preparation and characterization of coatings incorporated with poly (aniline-co-nitroaniline) nanoparticles having antifouling and anticorrosion behavior. Ind. Eng. Chem. Res..

[31] Brady R.F., Singer I.L. (2000). Mechanical factors favoring release from fouling release coatings. Biofouling.

